# Oxidative Stress Markers among Obstructive Sleep Apnea Patients

**DOI:** 10.1155/2021/9681595

**Published:** 2021-07-19

**Authors:** Agata Stanek, Klaudia Brożyna-Tkaczyk, Wojciech Myśliński

**Affiliations:** ^1^Department and Clinic of Internal Medicine, Angiology and Physical Medicine, Faculty of Medical Sciences in Zabrze, Medical University of Silesia, Batorego 15 St., 41-902 Bytom, Poland; ^2^Chair and Department of Internal Medicine, Medical University of Lublin, Staszica 16 St., 20-081 Lublin, Poland

## Abstract

Obstructive sleep apnea (OSA) is a chronic respiratory disorder, which can be present in up to 50% of the population, depending on the country. OSA is characterized by recurrent episodes of partial or complete obstruction of the upper airways with consistent movement of the respiratory musculature during sleep. Apneas and hypopneas can lead to a decrease in oxygen saturation, an increase in carbon dioxide in the blood, and subsequent arousals and sleep fragmentation caused by repetitive activation of the central nervous system. As a consequence, intermittent hypoxemia and consequent reoxygenation result in the production of reactive oxygen species, leading to systematic oxidative stress, which is postulated to be a key mechanism of endothelial dysfunction and increased risk for cardiovascular disorders in patients with OSA. In this review, various biomarkers of oxidative stress, including high-sensitivity C-reactive protein, pregnancy-associated plasma protein-A, superoxide dismutase, cell-free DNA, 8-hydroxy-2-deoxyguanosine, advanced oxidation protein products, lipid peroxidation products, receptor for advanced glycation end-products, and thioredoxin are discussed. Biomarkers of oxidative stress have the potential to be used to assess disease severity and treatment response. Continuous positive airway pressure (CPAP) is one of the most common noninvasive treatments for OSA; it keeps the upper airways open during sleep. This reduces episodes of intermittent hypoxia, reoxygenation, and arousal at night. CPAP has been shown to have anti-inflammatory properties and decrease oxidative stress. The administration of certain compounds, like vitamins A, C, and E as well as N-acetylcysteine and allopurinol, can decrease oxidative stress markers. However, their role in the treatment of OSA remains unclear.

## 1. Introduction

Obstructive sleep apnea (OSA), a chronic respiratory disorder, is present in up to 50% of the population, depending on the country, and affects nearly 1 billion adults aged 30–69 years [1]. OSA is characterized by recurrent episodes of partial or complete obstruction of the upper airways, with consistent movement of the respiratory musculature during sleep [2]. Apneas and hypopneas can lead to a decrease in oxygen saturation, an increase of carbon dioxide in the blood, and subsequent arousals and sleep fragmentation caused by repetitive activation of the central nervous system [3]. Frequent awakening during sleep is followed by somnolence during the day, lack of concentration, and chronic fatigue. The main risk factor for OSA is obesity; other risk factors include postmenopausal status in women, craniofacial dysmorphisms, alcohol use, overuse of hypnotics, and advanced age [4]. The risk of developing cardiovascular disorders such as ischemic heart disease, heart failure, arrhythmia, stroke, and transient ischemic attack is relatively high in patients with OSA [5, 6]. In addition, OSA can predispose patients to hypertension, irrespective of other factors. OSA may cause cognitive dysfunction as well and accelerate aging [7].

The first-choice method for diagnosing OSA is overnight polysomnography, which can assess the severity of OSA in terms of apneas and hypopneas per hour (AHI). OSA severity is divided into mild, moderate, and severe, depending on AHI per hour; mild patients have 5 to 14 episodes per hour, moderate cases have 15 to 29 per hour, and severe cases have over 30 per hour [8]. Additional parameters, such as oxygen desaturation index (ODI), measured as the mean number of oxygen hemoglobin saturation drops of 3% or more per hour of subjective sleep duration, mean blood hemoglobin oxygen saturation (SpO_2_), and duration of oxygen blood saturation below 90% (TSpO_2_ < 90%) may also be measured during polysomnography [9, 10]. The gold standard of treatment for OSA is a continuous positive airway pressure (CPAP), which can be delivered using a wide variety of devices. CPAP keeps the upper airways open during sleep and, as a consequence, reduces apneas and hypopneas [11].

## 2. Oxidative Stress in OSA

Recurrent episodes of disturbed airflow due to obstruction during sleep in patients with OSA can lead to apneas/hypopneas and sequent fluctuations in blood oxygenation, with intermittent hypoxemia and hypercapnia. As a consequence, intermittent hypoxemia and the consequent reoxygenation result in the production of reactive oxygen species (ROS), leading to systemic oxidative stress [12]. ROS can react with nucleic acids, proteins, and lipids leading to DNA alterations, cellular damage, and inflammation [6, 13]. Moreover, intermittent hypoxemia stimulates the production of proinflammatory factors and promotes metabolic dysregulation and platelet aggregation [14].

The systemic oxidative stress present in OSA may represent a key mechanism of endothelial dysfunction and a primary reason for the increased risk of cardiovascular found in this patient population [15, 16]. More specifically, ROS induce endothelial dysfunction in the early stages of OSA by increasing the expression of leukocyte-specific (L-selectin and integrins) and endothelial-specific adhesion molecules (E-selectin, P-selectin, ICAM-1, and VECAM-1). Additionally, endothelial dysfunction may also cause microvascular damage [17].

Sampol et al. first showed that patients who underwent thoracic aortic dissection had a high prevalence of previously undiagnosed and frequently severe OSA [18]. It was postulated that aortic dissection may relate to ROS production induced by intermittent hypoxia and hypoxia-inducible factor-1 (HIF-1). Moreover, the progression of aortic dissection is also affected by this pathway, which promotes the expression of vascular endothelial growth factor (VEGF), as well as matrix metalloproteinases 2 and 9 in the aortic wall [19].

Obesity, which occurs in more than 50% of OSA patients, is itself a chronic inflammatory state related to systematic oxidative stress and increased cardiovascular morbidity [6, 20]. Simiakakis et al. [21] showed that systemic oxidative stress in patients with OSA is not associated with disease severity but rather with the presence of obesity, smoking, and female sex.

### 2.1. Biomarkers of Oxidative Stress in OSA Patients

**Figure 1:**
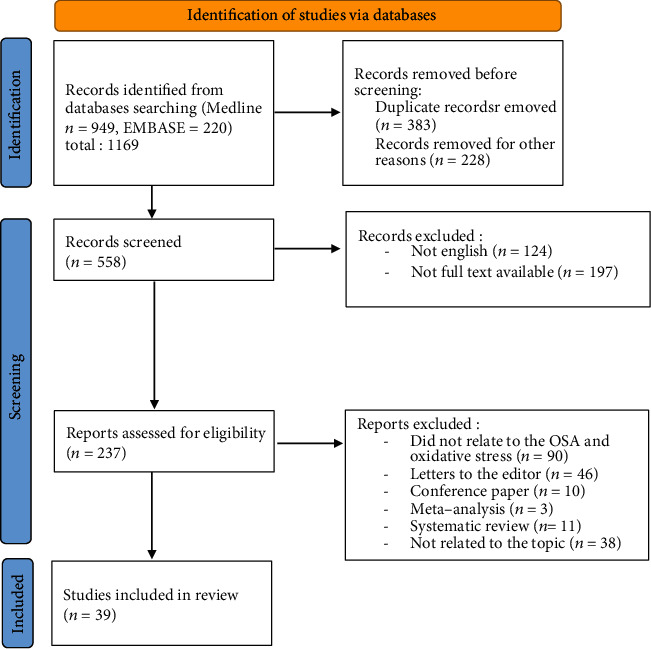
PRISMA flow diagram showing the study selection and identification.

In the following section, biomarkers of oxidative stress in patients with OSA will be discussed in more detail. For this purpose, Medline and Embase databases were queried, and only papers published in the last 20 years were analyzed. We used key words such as “obstructive sleep apnea,” “oxidative stress,” “CPAP,” and “antioxidants.” The present systematic review was reported based on the guidelines of the Preferred Reporting Items for Systematic Reviews and Meta-Analysis (PRISMA) Statement (Figure 1).

In our review, we only studied English articles, using systemic reviews, meta-analyses, prospective studies, and case reports. Biomarkers of oxidative stress in patients with OSA are presented in Table 1.

#### 2.1.1. High-Sensitivity C-Reactive Protein

A high-sensitivity C-reactive protein (hsCRP) is an acute-phase protein, in which high levels represent a marker of inflammation [22], though hsCRP may also be an oxidative stress marker [23]. There is a positive correlation between the levels of hsCRP and the parameters used to assess OSA severity, such as AHI, ODI, and SpO_2_ < 90% [24]. Obesity, assessed by body mass index (BMI), is a common comorbidity among OSA patients and is associated with increased oxidative stress, independently from presence of OSA [25]. While some view OSA as independent from obesity and elevated CRP levels [26], Volná et al. [24] reported a significant difference in the serum levels of hsCRP in patients without OSA compared to severe OSA after correction for BMI.

#### 2.1.2. Pregnancy-Associated Plasma Protein-A

Pregnancy-associated plasma protein-A (PAPP-A) belongs to the metalloproteinase family. PAPP-A is synthesized by the placenta during pregnancy; therefore, it is widely used as a marker for prenatal genetic screening [27]. Moreover, PAPP-A is produced by the colon, kidneys, endometrium, bones, and testicles, among other organs [28]. PAPP-A was found to be a marker of instability in atherosclerosis in coronary syndromes. Moreover, high levels of PAPP-A have been reported to be present in patients with asthma, chronic obstructive pulmonary disease, and lung cancer [29, 30]. Thus, a high level of PAPP-A may signal the presence of inflammation and oxidative stress and could be used as a biomarker of risk for patients with atherosclerosis [31–33]. Results from previous reports assessing the levels of PAPP-A in OSA patients remain ambiguous. Cengiz et al. [34] showed that PAPP-A levels were significantly higher in OSA patients compared with a control group. In this study, a negative correlation between AHI and the levels of PAPP-A was reported. Surprisingly, patients with moderate OSA were found to have higher levels of PAPP-A compared with those who had mild or severe OSA [34]. In contrast, Volná et al. reported no significant correlation between AHI and the severity of OSA [24]. The ambiguity of these results may be due to a relatively small study sample.

#### 2.1.3. Superoxide Dismutase Activity

Superoxide dismutase (SOD) is an essential antioxidant enzyme that eliminates ROS in a similar fashion to catalase and peroxidase. Lower activity levels of SOD were present in OSA patients compared with healthy subjects [35]. A significant reduction in SOD activity was reported in those who had mild to moderate OSA [13].

#### 2.1.4. Cell-Free DNA

High cell free-DNA (cfDNA) levels are considered a serum marker of many inflammatory diseases, such as stroke, ischemic heart failure, acute coronary syndrome, and OSA [21, 36, 37]. Free radicals in OSA patients destroy nucleic acids and lead to a higher level of free nucleosomes and cfDNA [38]. The levels of cfDNA were found to be significantly higher in OSA patients compared with healthy subjects [39]. Moreover, a linear correlation between cfDNA concentration and severity of disease was observed [40].

#### 2.1.5. 8-Hydroxy-2-deoxyguanosine

8-Hydroxy-2-deoxyguanosine (8-OHdG) is a product of DNA oxidation and has been used as a biomarker of oxidative stress. Significantly higher urinary 8-OHdG excretion has been observed in patients with severe OSA. In the same study, a positive correlation between 8-OHdG and AHI, ODI, and TSpO_2_ < 90% was observed [41]. In contrast, Jordan et al. [42] showed that the concentration of 8-OhDG correlates with a duration of lower saturation. However, urinary excretion remained within the normal range in OSA patients. Presumably, in this group, hypoxemia and consequent reoxygenation did not induce strong DNA damage.

#### 2.1.6. Advanced Oxidation Protein Products

Advanced oxidation protein products (AOPP) are a group of oxidized proteins that are produced by oxidation overload [43]. AOPP is a marker of both oxidative stress and inflammation [44]. They represent a more stable biomarker than products of lipid oxidation [45]. A study by Tóthová et al. [46] showed that AOPP concentrations in saliva samples are higher in the morning compared with the evening in patients with OSA. Thus, hypoxia, which occurs during sleep in OSA patients, induces oxidative stress. Yağmur et al. [47] discovered a positive correlation between AOPP blood concentrations and AHI, TSpO2 < 90%, and ODI. Moreover, higher levels of AOPP are observed in patients with severe and moderate OSA compared with those who had mild OSA or were healthy subjects. Mancuso et al. [48] published contradicting results, where there was no correlation between polysomnography parameters and AOPP concentrations.

#### 2.1.7. Lipid Peroxidation Products

End-products of lipid peroxidation include oxononenal (ONE), malondialdehyde (MDA), and hydroxynonenal (HNE). Lipid oxygenation is typically assessed by measuring the plasma concentrations of thiobarbituric acid-reactive substances (TBARS), which contain MDA and lipid peroxides. Hopps et al. [49] detected significantly higher levels of lipid peroxidation in patients with severe OSA compared with those who had mild and moderate disease severity. In all OSA patients (*n* = 48), positive correlations between AHI and TBARS and between ODI and TBARS were detected. Moreover, neck circumference, which is an anthropometric parameter used as a screening tool for obesity, was positively correlated with TBARS [49]. 8-Isoprostane is a product of the oxidation of arachidonic acid and is considered to be a reliable marker of oxidative stress because of its chemical stability [50]. Urinary excretion of 8-isoprostane was higher in OSA patients compared with healthy individuals [51].

#### 2.1.8. Receptor for Advanced Glycation End-Products

Advanced glycation end-products (AGE) are the results of the nonenzymatic glycation of proteins. This reaction can occur during intermittent hypoxia and hyperglycemia [52, 53]. Receptor for advanced glycation end-products (RAGE) is a multiligand cell-surface receptor that interacts with AGE. Interaction between the receptor and its ligand activates nuclear factor-kappa B (NF-*κ*B) and stimulates oxidative stress and inflammatory marker production. RAGE is expressed in podocytes, mesangial cells, and renal tubules, among other tissues [54]. Soluble RAGE (sRAGE) and endogenous secretory RAGE (esRAGE) were recently identified as two isoforms of this receptor. In OSA patients, sRAGE and esRAGE expression negatively correlated with AHI, ODI, and BMI [24, 55]. In addition, esRAGE expression relates to systolic and diastolic blood pressure; the lower the blood pressure, the higher the expression of esRAGE [55]. However, such a correlation was not found for sRAGE. Thus, it was suggested that these isoforms may have differing roles in OSA progression.

#### 2.1.9. Thioredoxin

Thioredoxin (Trx), along with NADPH and thioredoxin reductase (TrxR), is part of the Trx system, which is present in all living organisms. The main role of this system is to regulate a variety of cellular redox reactions and signaling pathways, such as antioxidant defense and gene transcription [56]. Guo et al. [57] found a positive correlation between the concentrations of Trx and the severity of OSA. Increased Trx levels were proportional to reduced O_2_ saturation and increased AHI values.

## 3. The Impact of CPAP on Oxidative Stress in OSA

CPAP is one of the most common noninvasive methods of treating OSA; by keeping the upper airways open during sleep, CPAP reduces episodes of intermittent hypoxia, reoxygenation, and arousal during the night. CPAP is also reported to decrease oxidative stress [58, 59]. The efficacy of CPAP depends on the duration of therapy. Alzoghaibi and BaHammam [60] showed that even one night of overnight CPAP treatment impacts lipid peroxidation and decreases TBARS concentrations. However, such a short therapy does not affect antioxidant production, and SOD activity remains unchanged after CPAP therapy. In contrast, decreased concentrations of 8-isoprostane from exhaled breathe were reported after only 2 days of CPAP therapy [61]. Likewise, three months of CPAP treatment led to a significant reduction in 8-isoprostane serum concentrations [58]. Borges et al. [62] reported that 8-week CPAP therapy had no significant impact on oxidative stress biomarkers, including SOD and AOPP. However, sleep efficiency and hours of sleep significantly improved. In several studies, AOPP concentrations remained unchanged after 3 months of CPAP therapy; however, the number of patients who underwent reevaluation after therapy was small (*n* = 7) [48]. Eight-week CPAP therapy also significantly decreased the levels of 8-OHdG [63]. With regard to Trx, studies have shown that one-month therapy significantly decreases the concentrations of this biomarker in patients with severe OSA [64].

## 4. The Role of Antioxidants in OSA

### 4.1. Vitamins and Microelements

Vitamins C, E, and A are essential microelements that act as antioxidants to protect lipids, proteins, nucleic acids, and other important biomolecules against oxidative stress damage [65, 66]. Their antioxidant properties are related to their ability to donate electrons. Selenium (Se), a metalloid, is present as selenoproteins, which are important in many reactions, such as the formation of thyroid hormones and antioxidant defense [67].

The role of antioxidants in OSA has been reported in many studies, though opinions may differ. Several studies reported decreased levels of vitamins A and E in OSA patients compared with healthy individuals [68, 69]. In contrast, Saruhan et al. [70] found that concentrations of vitamin A are increased in OSA patients and are positively correlated with AHI. The levels of vitamin E were also higher in OSA patients compared with healthy individuals, but the difference was not statistically significant, possibly due to the different sample sizes of the groups used in the study (47 vs. 114). Vitamins A and E, fat-soluble compounds, are stored in adipose tissue and the liver; oxidative stress and inflammation induce an increased release of vitamins from adipose tissue. Moreover, oxidative stress in OSA patients upregulates the expression of *α*-tocopherol transfer protein (*α*TTP), which is responsible for vitamin E transfer between the liver and other tissues [71]. However, the concentrations of vitamin C, a water-soluble compound, were significantly decreased in OSA patients compared with healthy subjects [70]. Opinions regarding the correlation between selenium levels and OSA also differ. Chen et al. [72] demonstrated that selenium concentrations are decreased in newly diagnosed OSA patients with mild to moderate severity. In contrast, Saruhan et al. [70] found significantly higher selenium levels in OSA patients compared with healthy subjects. In both studies, patients with comorbidities, which could impact the results, were excluded. The disparity between these studies could also result from varying sample sizes.

In the literature, several studies have investigated the use of antioxidants as therapy for OSA. Grebe et al. [73] performed measurements of flow-mediated dilation (FMD) on brachial artery by ultrasonography. FMD is a parameter that assesses endothelial function. FMD values were decreased in OSA patients; however, after intravenous administration of vitamin C, FMD increased in these patients, but not in the control group. In addition, animal models, including rats, have been used to assess the influence of vitamins C and E on oxidative stress. Intermittent hypoxia generated by obstruction of the trachea led to higher concentrations of MDA and AOPP, both indicators of oxidative stress. The administration of antioxidants significantly decreased AOPP levels, with no impact on MDA concentrations [59].

### 4.2. Medications and Perspective for Future OSA Therapy

Several medications frequently used to treat diseases other than OSA have antioxidant properties. NAC is well-known as a mucolytic drug and is used in acetaminophen intoxication. Moreover, NAC is essential to glutathione synthesis and has an antioxidant effect. A 30-day oral administration of N-acetylcysteine (NAC) has been previously tested in OSA patients. This study reported that in NAC-treated patients, lipid peroxidation products were significantly decreased and levels of glutathione were increased after treatment [13]. Allopurinol, which is commonly used in urate-lowering therapy, has additional properties, such as scavenging free radicals and inhibition of lipid peroxidation [74]. An animal model study, performed on rats, reported a significant decrease in lipid peroxidation products in allopurinol-treated rats [75].

## 5. Conclusion

Taken together, oxidative stress in patients with OSA arises as a consequence of intermittent hypoxia during the night. Biomarkers of oxidative stress may be used to assess disease severity as well as the individual response to treatment. Several of these markers, including high-sensitivity CRP, 8-hydroxy-2-deoxyguanosine, thioredoxin, advanced oxidation protein products, and lipid peroxidation, have positive correlations with AHI, ODI, and SpO_2_ < 90%.

CPAP therapy is essential to treat OSA due to its antioxidative properties. Many studies have reported a significant decrease in the concentrations of many oxidative markers, such as 8-isoprostane, 8-hydroxy-2-deoxyguanosine, and thioredoxin. The impact on the reduction of oxidative stress is greater the longer the therapy. However, SOD activity did not differ after the treatment. Likely, it is necessary to conduct further studies with a longer period of CPAP treatment and a wider study group.

In addition, surveys on the administration of antioxidants such as vitamins and antioxidant medications demonstrated decreased levels of markers of oxidative stress. However, several were performed on animal models, and further experimentation is warranted. Nonetheless, antioxidants may one day be utilized as complementary therapy for OSA, with potential benefits for patients who are intolerant to CPAP.

## Figures and Tables

**Table 1:** Characteristics of oxidative stress markers in obstructive sleep apnea (OSA) patients.

Author	Study population	Marker	Outcome
Volná J. et al. 2011 [24]	51 men divided into groups according to AHI values	hsCRP sRAGE PAPP-A	(i) Positive correlation between AHI, ODI, TSpO_2_ < 90%, and hsCRP levels (*p* < 0.001) (ii) Significant difference between hsCRP levels in severe OSA (AHI ≥ 30) compared to healthy individuals (AHI ≤ 5) (*p* = 0.039) (iii) Negative correlation between sRAGE and AHI (*p* = 0.044) and between sRAGE and ODI (*p* = 0.027) (iv) No correlation between OSA parameters and PAPP-A levels
Cengiz A. et al. 2018 [34]	44 OSA 44 control group	PAPP-A	(i) PAPP-A levels significantly higher in OSA, particularly in patients with moderate severity, compared to the control group (*p* < 0.001) (ii) Negative correlation between AHI and PAPP-A (iii) Positive correlation between minimum and mean oxygen levels at night and PAPP-A
Wysocka E. et al. 2008 [35]	41 OSA 39 control group	SOD	(i) Decreased activity of SOD in OSA compared to controls, particularly in those with moderate to severe severity (*p* = 0.006)
Bauça J. et al. 2017 [39]	62 OSA 52 control group	cfDNA	(i) Increased concentration of dsDNA in OSA compared to controls (*p* = 0.007)
Ye L. et al. 2010 [40]	127 OSA (43 mild, 39 moderate, and 45 severe) 52 control group	cfDNA	(i) Positive correlation between level of cfDNA and AHI and ODI (ii) Linear correlation between cfDNA concentration and severity of OSA
Yamauchi M. et al. 2005 [41]	75 OSA (17 nonsevere [AHI < 30], 58 severe [AHI ≥ 30]	8-OHdG	(i) Urinary excretion of 8-OHdG higher in severe OSA (*p* = 0.03) (ii) Positive correlation between 8-OHdG urinary excretion and AHI, ODI, and TSpO_2_ < 90%
Jordan W. et al. 2006 [42]	25 OSA (20 moderate to severe, 5 UARS or mild OSA)	8-OHdG	(i) 8-OHdG concentration slightly higher in patients with moderate to severe OSA (NS)
Tóthová L. et al. 2019 [46]	24 OSA (AHI > 30)	AOPP	(i) Salivary AOPP concentrations higher in the morning compared to evening (*p* < 0.05)
Yağmur A. et al. 2020 [47]	125 OSA (32 mild, 34 moderate, 59 severe) 40 control group	AOPP	(i) Higher levels of AOPP in severe and moderate OSA subjects compared to mild OSA subjects and healthy controls (*p* < 0.05) (ii) Positive correlation between AOPP blood concentration and AHI, TSpO_2_ < 90%, and ODI (*p* < 0.001)
Mancuso M. et al. 2012 [48]	41 OSA (7 mild, 15 moderate, and 19 severe) 32 control group	AOPP	(i) No correlation between AOPP levels and AHI
Hopps E. et al. 2014 [49]	48 OSA (21 low [AHI < 30], 27 high [AHI > 30])	TBARS	(i) Positive correlation between AHI, ODI, and TBARS concentration (*p* < 0.0001)
Cherneva R. et al. 2017 [51]	86 OSA (ESS < 11) 45 control group	8-Isoprostane	(i) Higher urinary excretion in OSA patients compared to controls (*p* = 0.028)
Cai W. et al. 2015 [55]	139 OSA (46 with AHI < 42, 46 with 42 ≤ AHI < 66, 47 with AHI ≥ 66)	sRAGE esRAGE	(i) Negative correlation between sRAGE and esRAGE expression and AHI (*p* < 0.05)
Guo Q. et al. 2013 [57]	54 OSA (14 mild, 11 moderate, and 29 severe)	Trx	(i) Positive correlation between AHI and Trx concentration (*p* < 0.05)
